# Poly(3-aminobenzoic acid) Decorated with Cobalt Zeolitic Benzimidazolate Framework for Electrochemical Production of Clean Hydrogen

**DOI:** 10.3390/polym12071581

**Published:** 2020-07-16

**Authors:** Kwena Desmond Modibane, Ngwako Joseas Waleng, Kabelo Edmond Ramohlola, Thabiso Carol Maponya, Gobeng Release Monama, Katlego Makgopa, Mpitloane Joseph Hato

**Affiliations:** 1 Nanotechnology Research Lab, Department of Chemistry, School of Physical and Mineral Sciences, University of Limpopo (Turfloop), Sovenga 0727, Polokwane, South Africa; ngwakowaleng@gmail.com (N.J.W.); kabelo.ramohlola02@gmail.com (K.E.R.); 2209thabiso@gmail.com (T.C.M.); release.monama@ul.ac.za (G.R.M.); 2 Department of Chemistry, Faculty of Science, Tshwane University of Technology (Acardia Campus), Pretoria 0001, South Africa; Makgopak@tut.ac.za

**Keywords:** poly(3-aminobenzoic acid), metal organic frameworks, electrocatalyst, hydrogen evolution reaction, Tafel analysis

## Abstract

A novel composite of poly(3-aminobenzoic acid) (PABA) and a cobalt zeolitic benzimidazolate framework (CoZIF) has been studied for the production of hydrogen through the hydrogen evolution reaction (HER). The structural characteristics and successful synthesis of PABA, CoZIF and the PABA/CoZIF composite were confirmed and investigated using different techniques. Probing-ray diffraction for phase analysis revealed that the composite showed a decrease and shift in peak intensities, confirming the incorporation of CoZIF on the PABA backbone via in situ polymerization, with an improvement in the crystalline phase of the polymer. The thermal stability of PABA was enhanced upon composite formation. Both scanning electron microscopy and transmission electron microscopy showed that the composite had a rough surface, owing to an interaction between the CoZIF and the external surface of the PABA. The electrochemical hydrogen evolution reaction (HER) performance of the synthesized samples was evaluated using cyclic voltammetry and Tafel analysis. The composite possessed a Tafel slope value of 156 mV/dec and an *α* of 0.38, suggesting that the Volmer reaction coupled with either the Heyrovsky or Tafel reaction as the rate determining step. The fabricated composite showed high thermal stability and excellent tolerance as well as high electroactivity towards the HER, showing it to be a promising non-noble electrocatalyst to replace Pt-based catalysts for hydrogen generation.

## 1. Introduction

Hydrogen gas (H_2_) is regarded as an alternative energy carrier for decreasing our dependency on fossil fuels (coal and oil) as well as reducing the greenhouse and other harmful gas emissions resulting from the energy processing of those fossil-fuel-based resources [1,2,3,4]. H_2_ gas can be produced by various methods, which include coal gasification, the steam reforming of natural gases and water splitting via photocatalysis or electrolysis [1,3]. Among these methods, electrochemical water splitting (EWS) (2H_2_O = 2H_2_ + O_2_) is the most promising and effective approach for achieving the abovementioned developments (coal gasification and steam reforming still produce greenhouse gases) [4]. During EWS, there are two important half-cell reactions taking place, viz., the hydrogen evolution reaction (HER) (4H^+^ + 4 e^−^ = 2H_2_) and oxygen evolution reaction (OER) (2H_2_O = O_2_ + 4H^+^ + 4e^−^) on the cathodic and anodic electrodes, respectively [1,2,5,6]. Both half-cell reactions require an electrode material that is highly electrocatalytically efficient, chemically and thermally stable and cost effective [6,7]. Currently, noble/precious transition metals including platinum (Pt), ruthenium (Ru) and palladium (Pd) are ideal HER electrocatalysts [1]. For example, Luo et al. [2] fabricated the Pt-based electrodes supported on Ru-Vulcan carbon (VC) for HER applications. The prepared Pt/Ru/VC electrocatalyst exhibited excellent HER activity as compared to commercial 20 wt.% Pt/C material. The Pt/Ru/VC electrocatalyst demonstrated good HER activity, including the lowest Tafel slope of 30.6 mV/dec, overpotentials of 23 and 30 mV to deliver current densities of 10 and 40 mA/cm^2^, and stability for 3000 cycles. In another study, Wang and co-workers [7] used an electro-filtration method to convert bulk Pt into a platinum single atom supported on a carbon, Pt-SAs/C HER electrocatalyst. The Pt-SAs/C electrocatalyst possessed high electrocatalytic HER activity with an overpotential of 36 mV to achieve a current density of 10 mA/cm^2^, a Tafel slope of 43 mV/dec and an outstanding stability up to 5000 cycles [7]. These metals are electrocatalytically active and stable, and have large cathodic current densities at lower overpotentials; however, high capital costs and scarcity greatly restrict their large-scale applications [1,6,8]. The quest for finding noble-metal-free and inexpensive HER electrocatalysts that exhibit similar activity with precious group metals is required. Conducting polymers (noble-metal-free), which are a group of organic polymers that possess an unusually high electrical conductivity (even in their doped form), offer potential as candidates for HER [9,10]. Polyaniline (PANI), which is one of the conducting polymers, and its derivatives have attained great attention due to their good electron density, ease of preparation and excellent environmental stability [10,11,12]. Furthermore, PANI derivatives have shown good processability, resulting from their improved solubility [12,13]. However, there are few reports on the use of polyaniline (PANI) and its derivatives as metal-free HER electrocatalysts. This is because the intrinsically conducting polymers (ICPs) are almost non-conductive in the potential window of the HER. Aydin and Koleli [14] reported the hydrogen evolution of PANI, polypyrrole (Ppy) and Ppy/PANI electrocatalysts deposited on a Pt surface. The results showed the HER followed a Volmer–Tafel process, where the Tafel step was the rate-limiting step. In addition, El-Deeb et al. [15] modified a glassy carbon electrode with PANI (GC/PANI) for HER applications in a Et_3_NHCl/[Bu_4_N][BF_4_]-CH_3_CN solution. The as-synthesized GC/PANI showed excellent HER properties as well as good stability. Previously, we reported on electrocatalytic HER studies of polyaniline and poly(3-amonobenzoic acid) homopolymers and their composites with metal organic frameworks (MOFs) [16,17]. 3-aminobenzoic acid (ABA) is one of the aniline derivatives with a carboxylic group on a meta- position to an amine group that can be polymerized to PANI derivatives such as poly(3-aminobenzoic acid) (PABA) by a similar radical mechanism as unsubstituted PANI [16]. From our studies, we observed that the electrocatalytic activities of the polymers were enhanced upon doping with metal organic frameworks [16,17]. The improved electrocatalytic activities in both studies were attributed to the increased electron density around the polymer backbone and additional active sites upon the introduction of MOFs, and the change in the structural properties of the materials, which led to improved kinetics for the charge transfer for the HER. Hence, we report for the first time the synthesis of a poly(3-aminobenzoic acid)/cobalt zeolitic benzimidazolate framework (PABA/CoZIF) composite as a proficient electrocatalyst for hydrogen production.

Zeolitic imidazolate frameworks (ZIFs), a subclass of MOFs, combine the superior properties of MOFs and zeolites [18,19]. These materials offer advanced properties such as a high surface area; unimodal micropores and permanent porosity; high crystallinity; abundant functionality; and excellent chemical, mechanical and thermal stability as compared to typical MOFs and zeolites [20,21,22,23,24]. Zhang et al. [25] reported ZIF-9 with an increased supercapacitance and improved electrocatalytic performance for oxygen evolution reaction (OER) in an alkaline electrolyte. They observed that ZIF-9 alone may be a desirable electrocatalyst for practical applications since at an overpotential of 0.362 V, it gave an exchange current density of 10 mA/cm^2^ and favorable OER kinetics with a Tafel slope of 68.35 mV dec^−1^. Unfortunately, some ZIFs suffer from the drawback of relatively poor stability, which would limit their practical applications for hydrogen production in acidic conditions. Therefore, combining PABA with ZIFs could offer great potential for the fabrication of an ideal HER electrocatalyst. The redox activity of the polymer would be retained due to the occurrence of protonation or deprotonation of the nitrogen atoms in the PABA backbone during the hydrogen evolution reaction.

## 2. Materials and Methods

### 2.1. Materials

Ferric chloride (FeCl_3_) (reagent grade, 97%) was obtained from Edu-chem Limited (Salford, UK). Tetrabutylammonium perchlorate (TBAP) (electrochemical analysis, ≥99.0%), ammonium persulfate ((NH_4_)_2_S_2_O_8_) (ACS reagent, ≥98.0%) and 3-aminobenzoic acid monomer (ABA) (98%) were purchased from Sigma Aldrich, South Africa. Ethanol (absolute, ≥99.8% (GC)), methanol (MeOH) (anhydrous, 99.8%), benzimidazole (C_7_H_6_N_2_) (98%) and cobalt nitrate hexahydrate Co(NO_3_)_2_.6H_2_O (99.99% trace metals basis) were all purchased from Merck, Germiston, South Africa. Dimethyl sulfoxide (DMSO) (ACS reagent, ≥99.9%), hydrochloric acid (HCl) (ACS reagent, 37%) and sulfuric acid (H_2_SO_4_) (ACS reagent, 95.0–98.0%) were acquired from Rochelle Chemicals, Johannesburg, South Africa. Distilled water was used to prepare all the solutions. All the reagents were used as obtained.

### 2.2. Synthesis of PABA, CoZIF and PABA/CoZIF Composite

#### 2.2.1. Fabrication of CoZIF

The CoZIF was prepared as in our recent study [21]. Briefly, about 3.0 g of benzimidazole and 0.520 g of Co(NO_3_)_2_·6H_2_O were dissolved in 40 and 30 mL of methanol, respectively. Both solutions were mixed and stirred for 24 h at room temperature. The resultant products were centrifuged and washed with ethanol three times then dried at 60 °C for 24 h.

#### 2.2.2. Preparation of PABA/CoZIF

**Scheme 1:**
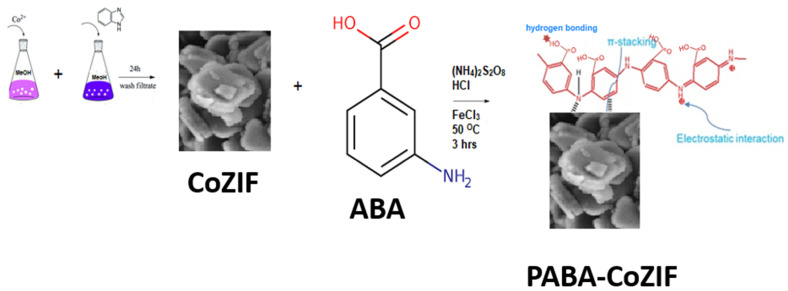
Preparation of poly(3-aminobenzoic acid) (PABA)/cobalt zeolitic benzimidazolate framework (CoZIF) composite.

The PABA/CoZIF composite (Scheme 1) was prepared by modifying the in situ chemical oxidation polymerization route [16,21]. Approximately, 1 g of the 3-aminobenzoic acid monomer and 0.036 g of ZIF-9 were dissolved in a solution of 10 mL of HCl and 100 mL of distilled water in a 250 mL round-bottom flask. The solution was stirred for 30 min at 50 °C. About 2.40 g of (NH_4_)_2_S_2_O_8_ and 1.88 g of FeCl_3_ were added into the solution. The reaction mixture was stirred for another 3 h at the same temperature. The mixture was placed in an oven overnight to evaporate the solvents, and the remaining content was washed with ethanol several times and re-dried at 50 °C. Consequently, the PABA homopolymer was synthesized as reported in our published work to be used for reference [16,26,27,28,29,30]. In addition, the PABA-to-CoZIF ratios were determined according to the work done by Al-Thani et al. [31].

### 2.3. Characterization Methods

To determine the functional groups and successful composite formation, the Spectrum II spectrometer (Perkin-Elmer, Johannesburg, South Africa) was operated at room temperature with a resolution of 4 cm^−1^ and a range of 4000–400 cm^−1^. The absorption studies were performed with the Varian Cary 300 UV-vis-NIR spectrophotometer (Agilent, Santa Clara, CA, USA), in the range 250–800 nm. The structures of PABA, CoZIF and the PABA/CoZIF composite were analyzed with the aid of X-ray diffraction (XRD, Phillips PW 1830, Eindhoven, The Netherlands) using the Cu-Kα radiation with λ = 1.5406 Å.

The thermal stabilities of the prepared materials were deduced using a simultaneous thermal analyzer (STA) 6000 from Perkin-Elmer (Johannesburg, South Africa), which was operated at a rate of 20 mL/min in purged N_2_ gas with heating from 30 to 500 °C at 10 °C/min.

The morphological characteristics of the prepared samples were determined by Auriga field emission scanning electron microscopy (FE-SEM), with an instrument from Carl Zeiss Microscopy GmbH (Jena, Germany) coupled with an energy dispersive X-ray spectrometer (EDS), and transmission electron microscopy (TEM) (FEI Tecnai G2 F20X-Twin MAT 200 kV Field Emission Transmission Electron Microscope) (Eindhoven, The Netherlands).

### 2.4. Hydrogen Studies

Electrochemical studies of PABA, CoZIF and PABA/CoZIF were carried out using a BASi Epsilon Electrochemical Analyser from Bioanalytical Systems Incorporated (West Lafayette, IN, USA) having a three electrode working system. Both the Pt working electrode and gold auxiliary electrode had a 3 mm diameter and an area of 0.071 cm^2^, and a Ag/AgCl electrode was used as the reference electrode. Electrochemical measurements were evaluated at 22 ± 2 °C, controlling the temperature with a water bath. The multiscans of the prepared samples (2.0 × 10^−4^ M) in 10 mL of 0.1 M TBAP/DMSO electrolyte solutions were acquired within a potential window ranging between −2.0 and 1.2 V with a scan rate of 0.02 to 0.10 Vs^−1^. The HER was performed in the prepared electrolyte employing CoZIF, PABA and PANI-CoZIF (2.0 × 10^−4^ M) as the electrocatalysts, using 0.033–0.450 M of H_2_SO_4_ as a source of hydrogen. The preparation of the H_2_SO_4_ standard solutions was done in 0.1 M TBAP/DMSO solution, used as a supporting electrolyte.

## 3. Results and Discussion

### 3.1. Characterization of Synthesized Materials

**Figure 1:**
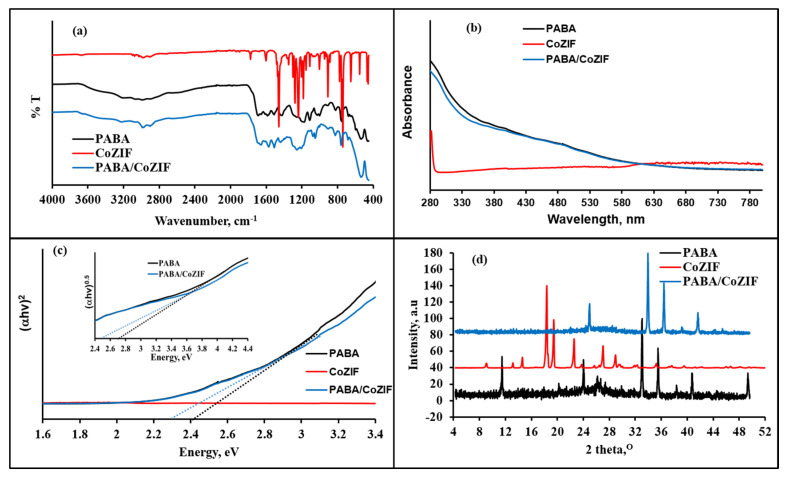
FTIR spectra (**a**); UV-Vis absorption spectra (**b**); Tauc plot for direct energy band gap (**c**) (inset: indirect energy band gap of PABA and PABA/CoZIF composite); and XRD patterns of synthesized PABA, CoZIF and PABA/CoZIF composite (**d**).

The FTIR spectra of CoZIF, PABA and PABA/CoZIF are shown in Figure 1a. The characteristic Co-N stretching band appearing at 3050 cm^−1^ confirms the interactions between Co^2+^ and nitrogen from the benzimidazole organic binder [21,32,33,34]. The FTIR spectrum of the PABA/CoZIF composite shows characteristics similar to those of neat PABA. The spectrum presents vibration bands around 807 and 1176 cm^−1^, which are attributed to the out-of-phase C-H bonds of benzenoid rings and the in-phase C-H bonds of quinoid rings, respectively [35,36]. The bands at 1337, 1505 and 1605 cm^−1^ are due to the presence of polyaniline derivatives, which consists of the C-N stretching of benzenoid and quinoid rings as well as the C=C of aromatic rings, respectively [36]. The band attributed to the *v*(C=O) that is uninterrupted during polymerization that appeared at 1700 cm^−1^ is due to the carboxylic acid group of PABA [37]. The broad band between 2500 and 3500 cm^−1^ is attributed to the N-H bond of the aminobenzoic acid [36]. Furthermore, there was no new peak after interaction with CoZIF, but the decrease in the strength of N-H absorption band upon doping is evident. This indicated the possibility of an interaction between PABA and CoZIF through electrostatic formation.

Figure 1b presents typical UV-vis spectra of PABA, CoZIF and PABA/CoZIF obtained from UV-Vis spectroscopy in DMF solvent. The spectrum of CoZIF shows a weak absorption band because of the limited absorption in the solar spectrum due to the relatively small extinction coefficients of Co^2+^ d-d transitions (~100–1000 mol L^−1^ cm^−1^) [38]. It can be observed that both PABA and the PABA/CoZIF composite have a broad absorption band between 600 and 300 nm. The similarities in the absorption bands are due to the π-π* transition of the benzenoid rings and exciton transitions of the quinoid rings in the PABA matrix [19]. Mashao et al. [21] reported that the photon absorption of polyaniline is found to obey the Tauc relation. Hence, in this work, Figure 1c shows the Tauc plots for PABA and the PABA/CoZIF composite according to Equation (1):

*αhν*(*v*) = *β*(*hv* − *E*_g_)*^n^* (1)

In this equation, *α*, *ν*, *β*, *h* and *E*_g_ represent the absorptivity coefficient, frequency of light, band gap tailing parameter, Planck’s constant and transition energy, respectively [21,33].

Furthermore, *n* in Equation (1) defines the transition probability index for direct allowed, direct forbidden, indirect allowed and indirect forbidden electronic transitions with n values of $\frac{1}{2}$, $\frac{3}{2}$, 2 and 3, respectively. The *E*_g_ were determined by an extrapolation of the linear part of the Tauc plot to where it cuts the abscissa using the direct allowed transition value of n = $\frac{1}{2}$. The *E*_g_ values were found to be 2.4 and 2.3 eV for PABA and the PABA/CoZIF composite, respectively. These findings are in line with the values reported in the literature [21,32]. This was further supported by indirect allowed transition energy values of 2.7 and 2.5 eV for PABA and the PABA/CoZIF composite, respectively. A decrease in the band gap energy of the PABA/CoZIF composite indicates an increase in the electron density at the polyaniline backbone [17].

To identify the phase structures of PABA, CoZIF and PABA/CoZIF powders in a 2θ range from 4° to 60°, XRD was used, and its patterns are presented in Figure 1d. It was seen that the diffraction peaks at 2θ angles of 6.98, 9.13, 13.03, 14.01, 15.44, 16.22, 18.73, 19.75, 21.14 and 23.04 correspond to the main peaks of crystalline CoZIF and were consistent with those reported by Zhang et al. [25] and Mashao et al. [21,33] with ICCD PDF #15-0806. A pattern showing a weak diffraction peak derived from crystalline Co was also observed at 2θ = 46°. On the other hand, the pattern for PABA shows the presence of many diffraction peaks with different intensities, confirming the nature of the copolymer as being polycrystalline [26]. The decrease and shift in the peak intensities in the pattern for the PABA/CoZIF composite confirms the incorporation of CoZIF on the PABA backbone via the in situ polymerization method, with an improved crystalline phase of the polymer.

**Figure 2:**
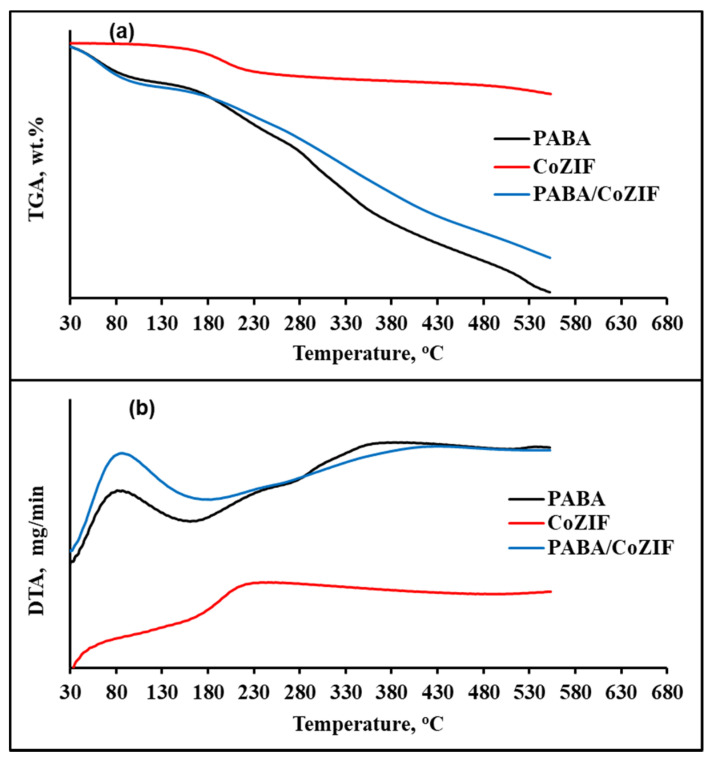
(**a**) Thermogravimetric analysis (TGA) and (**b**) differential thermal analysis (DTA) curves for PABA, CoZIF and the PABA/CoZIF composite.

Thermogravimetric analysis (TGA) and differential thermal analysis (DTA) are important techniques for determining the thermal stability as well as the exact weight ratio of each constituent that is present in a sample. TGA and DTA thermograms of all the samples (PABA, CoZIF and the PABA/CoZIF composite) are shown in Figure 2a,b, respectively. Figure 2a shows that CoZIF exhibits a degradation step, which appears at around 200 °C and was due to the loss of water, solvent and unreacted compounds [21,33,34]. This observation suggests that CoZIF was still thermally stable when the temperature was increased to 430 °C. Upon increasing the temperature to 550 °C, there is an observable slight weight loss, indicating a certain degree of structural decomposition of the ZIF framework [26]. ZIFs are a subclass of MOFs, and these materials normally exhibit two degradation steps at around 100 and 350 °C, which are due to the loss of moisture adsorbed in the framework of MOFs and disintegration of the organic linker, respectively [16,17]. Since CoZIF exhibited a less steep disintegration step, this observation suggests a high thermal stability of this material. This finding varies from the results previously reported for MOFs [4,21,33]. PABA has also been shown to be more thermally stable, but it was less stable than CoZIF. However, the thermal stability of PABA increased upon the incorporation of CoZIF due to the stabilizing effect of CoZIF, resulting in slower mass loss as compared to pristine PABA. Thus, an increase in the thermal stability of the PABA/CoZIF composite was observed compared to that of the neat PABA [28]. The enhanced thermal stability of the composite is attributed to the change in the morphology after the incorporation of CoZIF into the PABA matrix [29]. These transitions are clearly shown in the DTA results presented in Figure 2b. The figure shows an endothermic peak close to 100–150 °C on the DTA curve for PABA and the PABA/CoZIF composite, corroborating the loss of mass on the TG curve and the samples’ dehydration. A continuous mass loss was characterized in the interval of 180 to 380 °C on the TG curve, with two exothermic peaks showing maxima at 300 °C and 430 °C on the DTA curve, which correspond to the thermal decomposition of the polymer [17].

**Figure 3:**
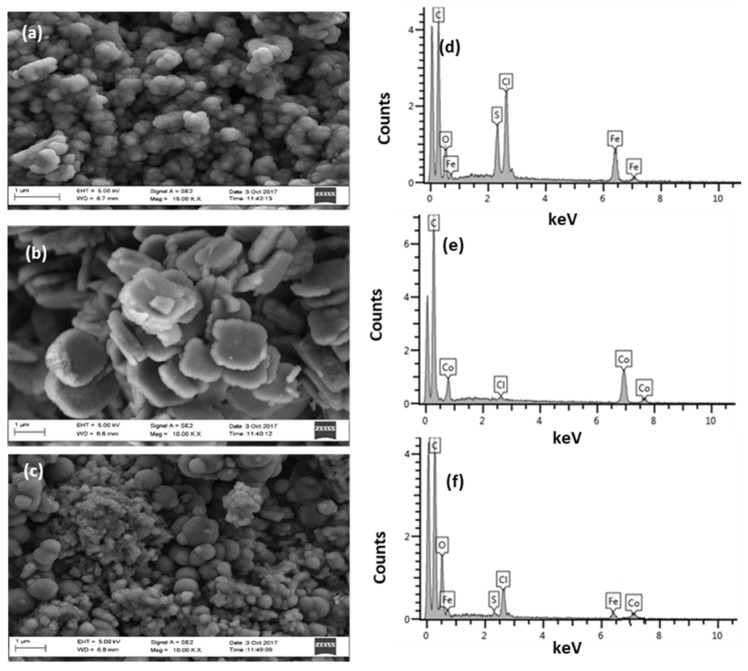
SEM images of (**a**) PABA, (**b**) CoZIF and (**c**) the PABA/CoZIF composite, and EDS spectra of (**d**) PABA, (**e**) CoZIF and (**f**) the PABA/CoZIF composite.

**Figure 4:**
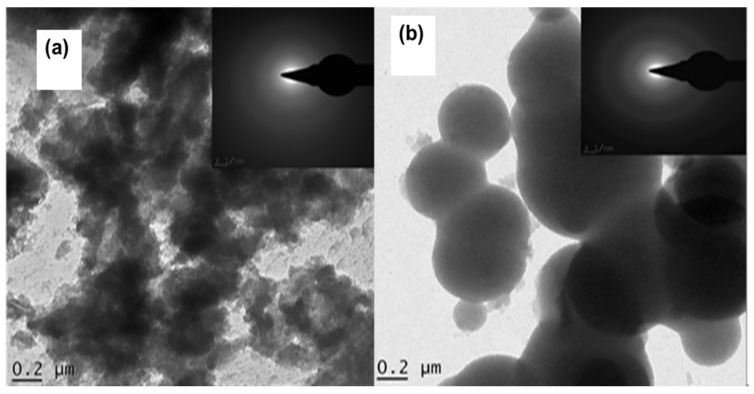
TEM images of (**a**) PABA and (**b**) the PABA/CoZIF composite; inset: selected area electron diffraction (SAED) images.

Figure 3 shows SEM images for PABA, CoZIF and PABA/CoZIF. The SEM image for PABA shown in Figure 3a depicts granular, spherical and hollow ball-like agglomerated morphology, while the CoZIF shows a typical tetrahedral or cake-like structure (Figure 3b). These morphologies are inconsistent with those reported in the literature [21,32,33]. Their corresponding EDS spectra are presented in Figure 3d,e, where their respective elemental compositions of carbon and cobalt can be observed. Upon the incorporation of CoZIF on the PABA backbone, there is a noticeable morphological change in PABA from spherical, granular and hollow ball-like to grain-like structures (Figure 3c) [30]. It is also interesting to note that there is a reduction in the particle size of the PABA upon composite formation, with some agglomeration [21]. The EDS results for the composite are given in Figure 3f. The percentage compositions of Co, C and O shown in Figure 3f are noticeable for the presence of CoZIF [21] and PABA [17], respectively. Moreover, the EDS analyses of PABA and the composite showed the presence of the Fe, Cl and S elements, which is due to the oxidant reagents used during the synthesis of the polymer and composite [4]. The SEM and EDS analyses showed that the PABA matrix is covering the CoZIF surface through non-covalent bonding between the reacting species [32,33]. The wrapping of PABA around CoZIF as observed in SEM was further confirmed by TEM, and the results are presented in Figure 4a for PABA and Figure 4b for the PABA/CoZIF composite, as the semi-crystalline nature of the CoZIF decreased upon incorporation [34,35,36]. The TEM image of PABA/CoZIF (Figure 4b) presents a highly disordered structure, confirming the fully amorphous nature due to the presence of PABA polymer [16], with the appearance of some spherical shape due to the presence of CoZIF [21]. The corresponding selected area electron diffraction (SAED) patterns (shown in the inset image) show no clear rings, which is indicative of an amorphous state, supporting the observed XRD result.

### 3.2. Electrochemical Studies

**Figure 5:**
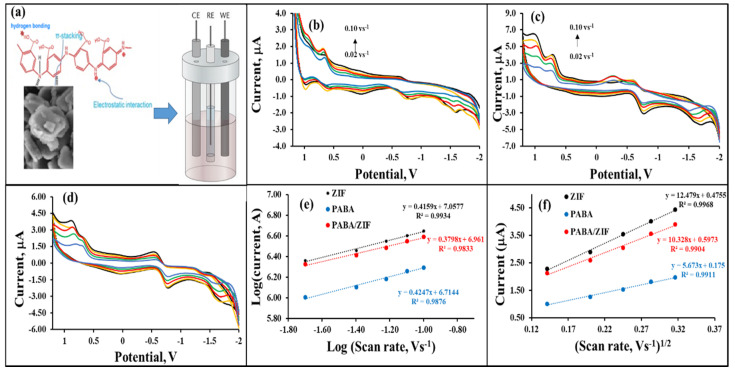
(**a**) Electrochemical set up; the cyclic voltammograms showing scan rate dependence for (**b**) Co-ZIF, (**c**) PABA and (**d**) the PABA/Co-ZIF composite; (**e**) the log of the current and scan rate curve; and (**f**) the Randles–Ševćik plots for the *I*_pa_ values for Co-ZIF (*E*_pa_ = 0.23 V), PABA (*E*_pa_ = 0.24 V) and PABA/CoZIF (*E*_pa_ = 0.20 V), using a Au electrode in a 0.1 mol.L^−1^ tetrabutylammonium perchlorate (TBAP)/DMSO electrolyte at scan rates of 0.02–0.10 V.s^−1^.

The PABA/CoZIF composite was characterized by cyclic voltammetry (CV, Figure 5a) at a scan rate of 0.1 V/s on a Au working electrode in a 0.1 M TBAP/DMSO solution. The CV results for the synthesized materials are presented in Figure 5b–d. The reduction and oxidation couples in all the materials at *Epc* ~ −0.6 and *Epa* ~ −0.8V are owing to the movement of ions from the TBAP/DMSO solution to the working electrode to establish the equilibrium of the system [37]. Upon the addition of CoZIF (Figure 5b), there is an increase in the current density in low potential regions, which agrees with the requirements of electrocatalysts [16]. Upon the introduction of PABA as shown in Figure 5c, the voltammogram showed a proliferation of cathodic and anodic current, with an increase in the scan rate. Moreover, the existence of carboxylic groups on the polyaniline derivative backbone produces a voltammogram that is different from that of a neat polyaniline [16,21,38]. The PABA/CoZIF composite, as shown in Figure 5d, showed a shift in and an improvement of cathodic and anodic current peaks at *E*_pa_ = 0.20 V with reference to neat PABA (*E*_pa_ = 0.24 V). It indicates that the synergetic effects of the PABA and CoZIF components contributed to the conductivity of the composite and that electrons were able to diffuse through the polymer chain [39]. These voltammograms show a quasi-reversible process [4,16,21,33] in which current densities increase with an increase in scan rates, with little shift in overpotentials towards lower values. The increase in current density is indicative of an electric-charge-transfer-controlled process in the composite and also shows that the electron conduction had increased upon doping [4]. We speculate that the benzene ring of CoZIF can provide recognition sites through a “π–π stacking” interaction with the aromatic structure of PABA. Therefore, Co-ZIF may play an important role of enhancing the PABA electrocatalyst to improve its sensitivity and stability properties.

The dependence on the scan rate in the voltammograms for PABA, CoZIF and PABA/CoZIF is presented in Figure 5e,f. The logarithmic plots (Figure 5a) and Randles–Ševćik plots (Figure 5e) for the *I*_pa_ values of the neat PABA and PABA/CoZIF composite are displayed together, as is the one for CoZIF. The linearity of the plots in Figure 5f indicates that the PABA and CoZIF materials in the final composite possess a charge transfer ability along the polymer backbone [4,5,16,17]. The diffusion coefficients, *D* (cm^2^ s^−1^), of the synthesized materials represented by the slopes of the Randles–Ševćik curves were determined using Equation (2) [17].

*I*_p_ = (2.65 × 10^5^)*n*^3/2^*ACD*^1/2^*v*^1/2^ (2)

The *v*, *C*, *n*, *A* and *I*_p_ symbols represent the scan rate (Vs^−1^), concentration of the catalyst (mol cm^−3^), number of transferred electrons, working electrode area (cm^2^) and peak current (A), respectively. Correspondingly, the *D* values were found to be 1.39 × 10^−7^, 4.15 × 10^−7^ and 3.23 × 10^−7^ cm^2^ s^−1^ for CoZIF, PABA and PABA/CoZIF, respectively, and are consistent with those reported in the literature for polymers doped with MOFs [32,38,40].

### 3.3. Hydrogen Evolution Reaction Studies

**Figure 6:**
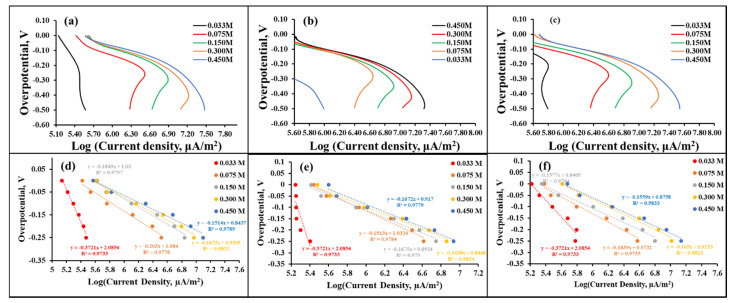
Tafel plots for (**a**) PABA, (**b**) CoZIF and (**c**) the PABA/CoZIF composite, and typical linear polarization curves from the Tafel plots for (**d**) PABA, (**e**) CoZIF and (**f**) the PABA/CoZIF composite in the presence of 0.033–0.450 M H_2_SO_4_ at 0.10 V s^−1^ on a Au electrode in 0.1 M TBAP/DMSO electrolyte.

To find the overpotential required for hydrogen evolution on the catalyst surface, Tafel analysis was performed using H_2_SO_4_ in 0.1 M TBAP/DMSO on a Au electrode. The Tafel curves are presented in Figure 6a–c for PABA, CoZIF and the PABA/CoZIF composite, respectively. It was reported that an increase in current density (in terms of shift in logarithm current) in the presence of an increase in the H_2_SO_4_ concentration is an indicator of the HER [40,41]. The intrinsic properties of electrocatalysts together with their catalytic efficiencies are usually studied using Tafel plots [40,41,42,43,44]. The linear polarization curves are presented in Figure 6d–f for PABA, CoZIF and the PABA/CoZIF composite, respectively. The parameter *b*, representing the Tafel slope, was obtained by fitting the Tafel plot (Figure 6d–f) to the Tafel equation (*ɳ* = *b* log (*j*) + *a*), and the charge transfer coefficient, *α*, was determined according to the Tafel equation (*b* = −2.303 RT/(1-α)F). The *b* and *α* are important parameters that can provide insight into the HER mechanism on the investigated electrocatalyst [16,17], and their data are given in Table 1. The proposed HER mechanism in the acidic medium is given by the three major reactions shown in Equations (3)–(5) [17].

Firstly, the adsorption of the hydrogen proton on the surface of an electrocatalyst results in a Volmer reaction [16]:

(3) $$H^{+}+e^{-}\to H_{\mathrm{ad}},\;b=\frac{2.3RT}{\alpha F}\approx 120\;\mathrm{mV}{\mathrm{dec}}^{-1}$$

where *T* and *R* stand for the thermodynamic temperature (K) and gas constant (8.31451 J mol^−1^ K^−1^), respectively. α≈ 0.5 is the symmetry coefficient, and *F* is Faraday’s number (96,485 C mol^−1^) [1]. This is followed by either a reaction between an adsorbed hydrogen and a proton from the electrolyte (electrochemical desorption or Heyrovsky reaction) [4]

(4) $$H_{\mathrm{ad}}+H^{+}+e^{-}\to H_{2},\;b=\frac{2.3RT}{\left(1+\alpha \right)F}\approx 40\;\mathrm{mV}{\mathrm{dec}}^{-1}$$

or a reaction between two adsorbed hydrogen protons next to each other to give molecular hydrogen [21,33]:

(5) $$H_{\mathrm{ad}}+H_{\mathrm{ad}}\to H_{2},\;b=\frac{2.3RT}{2F}\approx 30\;\mathrm{mV}{\mathrm{dec}}^{-1}$$

Table 1 shows a comparison of the calculated slopes of the PABA/CoZIF with that reported in the literature. The gold electrode (blank) gave high Tafel slopes of 459.3 and 471.4 mV dec^−1^ at 0.0300 M and 0.150 M H_2_SO_4_ concentrations, respectively. The slope decreased upon the addition of PABA, CoZIF and the PABA/CoZIF composite on the surface of the electrode as compared to that with the Au electrode [32,45,46]. The obtained *b* values were in the range of 150–373 mV dec^−1^, differing according to acid concentration as shown in Table 1. The high *b* values show that the rate-determining step is the Volmer step, and production of molecular hydrogen follows the Volmer step coupled with either the Heyrovsky or Tafel step [4,16,21]. Furthermore, this was supported by the charge transfer coefficient, which was determined to be closer to 0.5 [16]. The intrinsic activity of the electrocatalyst was further estimated by determining the exchange current density, *j_o_*, by extrapolating the Tafel plots [21,33]. The *j_o_* value obtained for the synthesized PABA/CoZIF was greater than that for the pristine PABA and CoZIF at lower concentrations, showing it to be a promising electrocatalyst for hydrogen production.

## 4. Conclusions

In summary, we have successfully developed a PABA/CoZIF composite electrocatalyst from the incorporation of CoZIF in PABA polymer backbone through electrostatic interaction. The electrocatalytic efficiency of the synthesized PABA/CoZIF composite for hydrogen production was investigated in an acidic medium through the HER. Both SEM and TEM demonstrated that the composite had a rough surface due to an interaction between the CoZIF and the external surface of the PABA. The composite exhibited admirable catalytic action due to the presence of CoZIF and remarkable stability for the HER in the acidic medium as compared to pristine PABA. Moreover, the electrocatalytic activity of the PABA/CoZIF composite appeared to be greater than that of CoZIF and PABA. The synthesized composite possessed a Tafel slope value of 156 mV/dec and charge transfer coefficient of 0.38, indicating that the rate-determining step of the HER for PABA/CoZIF was the Volmer reaction coupled with Heyrovsky reaction or Tafel reaction. This study affords a route for preparing a PABA doped with CoZIF electrocatalyst with high competence and incredible electrocatalytic action for the HER.

## Figures and Tables

**Table 1:** Hydrogen evolution reaction (HER) parameters for the PABA, CoZIF and PABA/CoZIF electrocatalysts.

Material	H_2_SO_4_ (mol L^−1^)	Slope (*b*) (V dec^−1^)	−*b* (mV dec^−1^)	1−*α*	Log (*j*_0_/μA m^−2^)	*j*_0_ (A m^−2^)
Blank	0.033	−0.4593	459.3	0.1288	5.88	0.76
	0.15	−0.4714	471.4	0.1255	5.88	0.76
PABA	0.033	−0.3721	372.1	0.1589	5.93	0.85
	0.075	−0.203	203.0	0.2913	6.22	1.66
	0.15	−0.1849	184.9	0.3198	6.39	2.45
	0.3	−0.1673	167.3	0.3535	6.70	5.01
	0.45	−0.1514	151.4	0.3906	7.03	10.72
CoZIF	0.033	−0.3721	372.1	0.1589	5.95	0.89
	0.075	−0.1913	191.3	0.3091	6.20	1.58
	0.15	−0.1675	167.5	0.3531	6.54	3.47
	0.3	−0.1638	163.8	0.3610	6.73	5.37
	0.45	−0.1672	167.2	0.3537	6.95	8.91
PABA/CoZIF	0.033	−0.3721	372.1	0.1589	6.07	1.17
	0.075	−0.1837	183.7	0.3219	6.24	1.74
	0.15	−0.1577	157.7	0.3750	6.52	3.31
	0.3	−0.165	165.0	0.3584	6.81	6.46
	0.45	−0.1559	155.9	0.3793	7.00	10.0
Pd@CuPc/MOF on Pt electrode [25]	0.300	−0.177	177.0	0.3341	6.95	8.91
20% Pt/C on GCE electrode [2]	0.500	−0.0306	30.6	1.9326	-	-
Ru/VC on GCE electrode [2]	0.500	−0.0867	86.7	0.6821	-	-
Pt/VC on GCE electrode [2]	0.500	−0.1328	132.8	0.4453	-	-
Pt/Ru/VC on GCE electrode [2]	0.500	−0.0306	30.6	1.9326	-	-

CuPc = copper phthalocyanine; GCE = glassy carbon electrode; VC = Vulcan carbon.
